# Effects of the Insertion Type and Depth on the Pedicle Screw Pullout Strength: A Finite Element Study

**DOI:** 10.1155/2018/1460195

**Published:** 2018-07-26

**Authors:** K. Jendoubi, Y. Khadri, M. Bendjaballah, N. Slimane

**Affiliations:** ^1^École Nationale Supérieure d'Ingénieurs de Tunis, Tunis, Tunisia; ^2^Laboratoire de Biomécanique, Institut National d'Orthopédie M.T. Kassab, Tunis, Tunisia; ^3^Biomedical Technology Department, King Saud University, Riyadh, Saudi Arabia

## Abstract

**Purpose:**

The pedicle screw is a surgical device that has become widely used in spinal fixation and stabilization. Postsurgical complications such as screw loosening due to fatigue loading and screw breakage still need investigations. Clinical parameters such as the screw insertion type and depth, the bone density, and the patient degree of mobility greatly affect the mechanisms of the implant's failure/success.

**Methods:**

The current finite element study focused on the prediction of the pedicle screw pullout strength under various conditions such as insertion type, insertion depth, bone quality, and loading mode.

**Results:**

As depicted in this study, the preservation of the pedicle cortex as in the N1 insertion technique greatly enhances the pullout resistance. In addition, the higher the screw-anchoring depth, permitting to gear a maximum number of threads, the better the protection against premature breakouts of pedicle screws.

**Conclusions:**

In agreement with experimental data, the type of insertion in which the first screw thread is placed immediately after the preserved pedicle cortex showed the best pullout resistance for both normal and osteoporotic bone.

## 1. Introduction

Spinal surgery has been remarkably improved by using pedicle screws. However, the use of such a successful surgical tool can achieve its objectives only by means of theoretical knowledge and rigorous practice. The critical location of the screws near the main nerve roots makes these elements very delicate in spine surgery causing serious and traumatic postoperative complications to the patient when inserted inaccurately [1].

Although the penetration of bony landmarks is reliable, the demands for multistage instrumentation require adjustments that necessitate an extensive and practical knowledge of spinal morphology [2]. The rate of pullout for pedicle screws, which indicates the in vivo rate of failure/success of the fixation devices, was investigated in few follow-up studies. According to Mayo et al. [3], the estimated rate of pullout for spine pedicle screws is about 10 to 15%. The number of published works in this field remains, however, insufficient and very limited. In an earlier study [4], conducted on 66 patients with instrumented spinal hardware “Zielke,” 12 cases of pullout were identified among which only one suffered from a loss of correction. In a survey analysis of a series of 617 surgical cases [5], pullout rates ranging from 0.6 to 11% were recorded depending on the age of the treated patients. Among the tested cases, 2.9% of the used pedicle screws were sharp broken before the end of the treatment.

Moreover, several observed cases showed through standard radiographs that the implanted screws can have not only diverse trajectories but variable depths of penetration as well. Due to excessive mechanical stress, some of these screws may have undergone premature failure in vivo. Sterba et al. [6] have addressed the biomechanical stability of the angled screw insertion. In their experimental study, screws were angled into the pedicles and parallel to the spinous process prior to analyzing total damage during cyclic loading. Their results showed that significant stability was achieved in straight screws compared with the angled ones [6, 7].

From a biomechanics perspective, a scoliotic spine will exert pullout forces on the pedicle screws that tend to extract them from their anchorage. These pullout efforts vary in terms of amplitude and rate of loading depending upon the patient's mobility. They might be either slow, low in magnitude leading to static-loading conditions, or fast and high in magnitude as in the case of dynamic- or impact-loading conditions. The consequence of these loading conditions on the bone at the vicinity of pedicle screw anchorage is devastating, especially in the early months of stabilization. The need to study the bonding mechanisms between the pedicle screws and vertebra requires a close collaboration between orthopedic surgeons and engineers [8].

The treatment of certain spinal injuries requires a clear vision about the surgical procedure and the hardware shape and materials to be adopted. In practical terms, the surgeon must first identify the access area on the posterior aspect of the vertebra, the screw insertion angles in both sagittal and transverse planes, and finally the shape of the screw to be selected and to which depth it has to be inserted.

All these facts and parameters show the importance that pedicle screws can play in spinal surgery and justify, as well, the interest of this research in terms of simulation and modeling. For this reason, the present study focused on the development and use of 2D axisymmetric finite element models to compute the pullout efforts acting on the pedicle screws in spinal surgeries as well as the stress state in the immediate vicinity of the implants for different configurations of screw insertion levels [9]. These parameters, tackled by several authors [10], are known to be some of the most vital parameters that govern the stability and endurance of the spinal implants. All the computational analyses were conducted for both configurations of the bone density, normal bone, which is a characteristic of healthy young subjects, and osteoporotic bone, usually seen in elderly people [11, 12].

The stress distribution in the bone was predicted and expected to help understand the mechanical behavior of the screw during the tearing process, under the influence of the abovementioned geometric, physiological, and surgical parameters [13–15]. Lughmani et al. [12] proposed a method to investigate bone quality upon which their experimental results showed that the drilling force does not only vary from one animal bone to another but does also vary within the same bone due to its changing microstructure.

## 2. Materials and Methods

In order to generate the 2D computer-assisted design (CAD) model, a portion of the vertebra and the screw was isolated at the screw centerline level in order to keep the axis of symmetry of the screw, through which the pullout efforts are transmitted. This permits abridging a complex 3D structure into a simpler 2D axisymmetric model that is accurately representative of an adult lumbar vertebra.

### 2.1. Geometric Considerations

**Figure 1:**
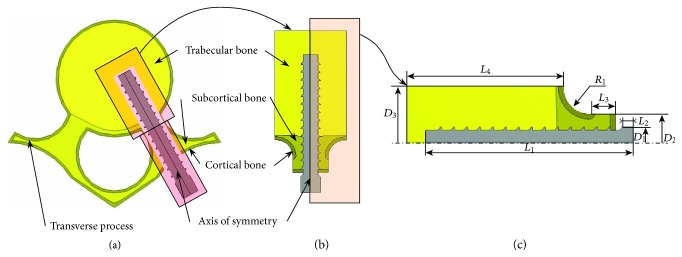
Extraction of a 2D axisymmetric model for the screw and the surrounding bone structures: (a) global model of the vertebra and pedicle screw, (b) boundaries of the axisymmetric model, and (c) relevant dimensions used in the development of the 2D axisymmetric model.

To reflect the multiscale aspect of the bone, a partition into three areas was made during the model development (see Figure 1(a)). This consideration was helpful in the implementation of different mechanical properties for the cortical, subcortical, and trabecular bone structures [16]. In all model investigations, the cortical bone thickness was set equal to one (1) millimeter [17]. It is to be noted that the pedicle is made only of cortical and subcortical bone tissues [18].

While Figure 1(b) shows the boundary of the bone domain commonly called the area of influence where the bone is affected by the implant's tearing and loosening, Figure 1(c) describes and sets all parameters pertaining to the development of the bone and screw axisymmetric model. Those parameters are fully described and listed in Table 1. In order to make the most of the axisymmetric geometry at the vertebral level, we proceed to the virtual removal of the vertebral transverse process while retaining its effect in the form of coupling equations implemented during the computational works; this issue is discussed in more details later on in the manuscript.

**Figure 2:**
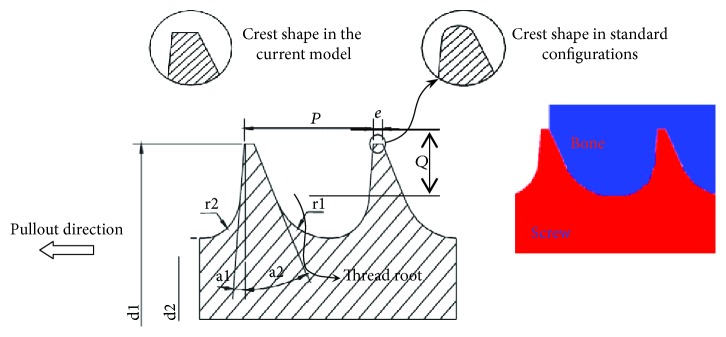
Detailed screw parameters associated with the 2D axisymmetric CAD model.

The screws used in orthopedic surgery have very intricate shapes and dimensions [19]. The major dimensions for the pedicle screw shown in Figure 2 were set according to the British standards [20] and are listed in Table 2.

The detailed CAD model for the pedicle screws and surrounding bone was developed using Abaqus/CAE preprocessor. Precautions were taken into account while developing the axisymmetric model, particularly at the junction between the pedicle screw and the vertebra body, to accurately capture the stress state generated by the loading. In order to optimize the FE mesh, the round fillets at the top of the standard crests were eliminated and replaced by sharp edges (see Figure 2). It is worth mentioning that the axisymmetric model is generated by revolving the profile of the threads around the screw centerline, leading to material discontinuity between two adjacent threads.

### 2.2. Stress-Strain Relationship for Vertebral Bone Structures

The mechanical behavior of the human vertebral cortical, subcortical, and trabecular bone structures is described in several studies [16, 21]. To simulate high loading conditions leading to bone failure, we opted for an elastic-plastic behavior that describes the stress-strain relationships in the elastic zone and perfect plastic zone which end up in abrupt failure. All the parameters associated with the stress-strain relationship for the cortical, subcortical, and trabecular bone structures, in their normal and osteoporotic conditions, are grouped in Table 3. Young's modulus (*E*) and the plastic yield stress (*σ*_p_) and strain (*ε*_p_) were deduced from density values based on empirical relations. Also listed in this table are the mechanical properties of the titanium alloy used for the pedicle screws.

### 2.3. Mesh Generation and Boundary Conditions

**Figure 3:**
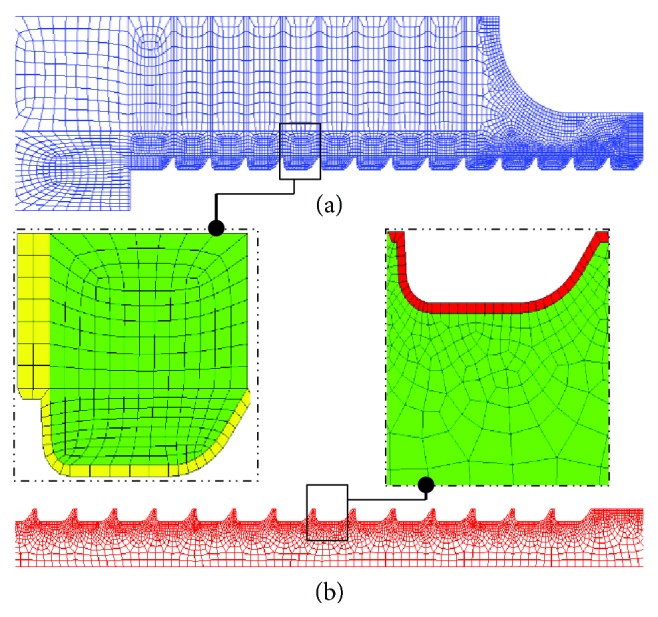
A closer view of the refined mesh characteristic of the interface between the bone (a) and screw (b).

The discretization process of the model passed through critical steps prior to mesh generation. A preliminary investigation was performed to find the optimal mesh parameters for each of the partitions shown in Figure 1. That is, in this phase, a minimal number of elements and nodes were sought to achieve appropriate convergence of the computed results. The grid density was then increased to account for higher stress gradient occurring at the screw-bone interface, an intricate area responsible for the load transfer (see Figure 3(a)).

The linear axisymmetric 4-node quadrilateral elements were preferred to their linear triangular counterparts due to their ability in producing regular meshes on both sides of the interacting surfaces. Such a regular and identical mesh at the screw-bone interface for the various models permits a better comparison platform of the computed results. A mesh refinement characterized by a minimal element size of 0.1 mm was implemented in such areas, leading to a total number of nodes and elements for all models of around 28,000 and 27,000, respectively.

By their morphological aspect, highly porous vertebrae raise a problem when it comes to computing their contact stresses and load transfer mechanisms while being in contact with an extremely stiffer material, namely, titanium. Special attention was focused on modeling the contact at the screw-bone interface. A free surface-to-surface contact was adopted for such an interaction with nodes on the softer bony area playing the role of slave nodes while their counterpart on the harder screw were set to be master nodes. For all conducted analyses, either in small or large deformation formulations, the friction coefficient between the two surfaces was set to 0.2 [18].

To simulate a tensile-loading condition, a prescribed displacement was imposed on the screw head via a multipoint constraint (MPC) coupling condition. The coupling condition consists of selecting the nodes on the flat area of the screw head to be coupled to a reference point (RP) located on the screw centerline to which a movement is prescribed to reproduce the tensile test. To get closer to the ultimate conditions that supposedly lead to implant failure, large deformation formulation was made active in Abaqus explicit solver, and trial loadings were carried out to establish the amount of load that induces large deformations on the bone structure. A 500 *μ*m shift applied to the RP at a rate of 2 mm/min was found to trigger such behavior and was adopted for the future large deformation analyses. On the opposite, using the default Abaqus standard solver, a 10 *μ*m shift on the RP was selected for small deformation situations to simulate static, low-magnitude loading conditions. An MPC equation was set to account for the material continuity of the vertebral transverse process and ensure the transmission of the cohesive forces. Finally, a fixed boundary condition was set on the outermost boundary while symmetry boundary was administrated to nodes on the model's axis of symmetry (see Figure 3(b)).

### 2.4. Screw Insertion Techniques

The failure mechanisms are known to be most active at the bone-screw interface. Several scenarios can schematically predict the mechanisms of bone failure leading to implant pullout. To help elucidate the failure mechanism in bony structures, the von Mises and shear stresses developed due to small deformation and large screw excursions were thoroughly investigated.

**Figure 4:**
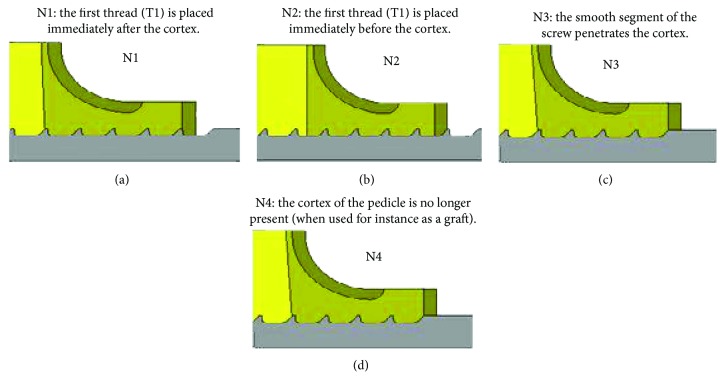
CAD models of four screw insertion types considered in the present study.

The most common screw insertion techniques adopted by surgeons in spinal fixation surgeries were modeled in the current study to analyze their respective effects on the stability of pedicle screws when implanted in the vertebral body.  Figure 4 defines and graphically illustrates each of the selected models.

### 2.5. Influence of the Number of Threads Engaged on the Pullout Solicitations on the Screw

Pedicle screws have, in general, standard shapes and sizes; they are fully or partially implanted during surgery, depending on the spine segment to treat, the trajectories of access to the anchorage sites, and the quality of bone (osteoporotic or normal). To mimic this situation, five depths of implantation characterized by a number of 2, 5, 8, 11, and 14 engaged threads were modeled for the N1 insertion type. Mechanically, this is equivalent to modifying the contact stress at the bone-implant interface, affecting by the same fact, the overall tension and tearing solicitations imposed on the implant.

## 3. Results and Discussions

### 3.1. Influence of the Screw Insertion Type on the Stress Distribution in the Surrounding Structures

**Figure 5:**
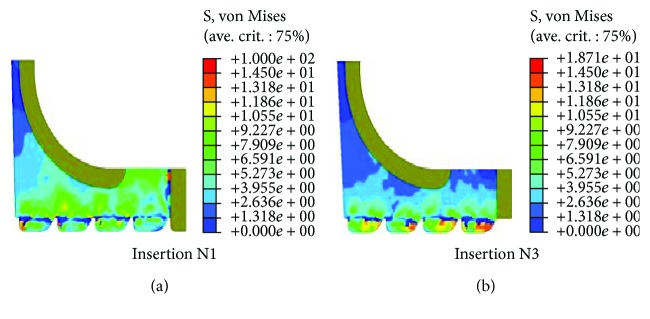
von Mises stress distribution in normal pedicle subcortical bone for the last loading step. Results were computed for the two insertion types of N1 (left) and N3 (right) using large deformation formulation.

Since it is one of the most broadly used techniques in surgery, the “N1” insertion type was selected for the analysis against the “N3” type, expected to play a less resistive role in screw pullout situations.  Figure 5 tracks the von Mises stress distribution along the screw-bone interface, computed as a response to a large excursion of the pedicle screw using a large deformation formulation. The highest stresses took place in the tiny bony anchorage sites opposite to the screw threads placing, therefore, the implant at high risk of failure. Thin blue filaments at the vicinity of the screw threads indicated that the bone in such areas had displayed a full range of plastic deformation before total failure.

The stress distribution portrayed in the subcortical bone for the last loading step (500 *μ*m) clearly shows the high von Mises stress value of about 100 MPa raised in the pedicle subcortical bone for the N1 insertion, in which the first thread presses against the cortical wall impeding the screw-tearing mechanism. Conversely, the deep placement of the screw in the N3 insertion usually breaks the cortical wall leading to a more active pulling mechanism. The von Mises stresses induced in the subcortical bone, for instance, increased by almost five folds when the N1 is preferred to the N3 insertion type.

**Figure 6:**
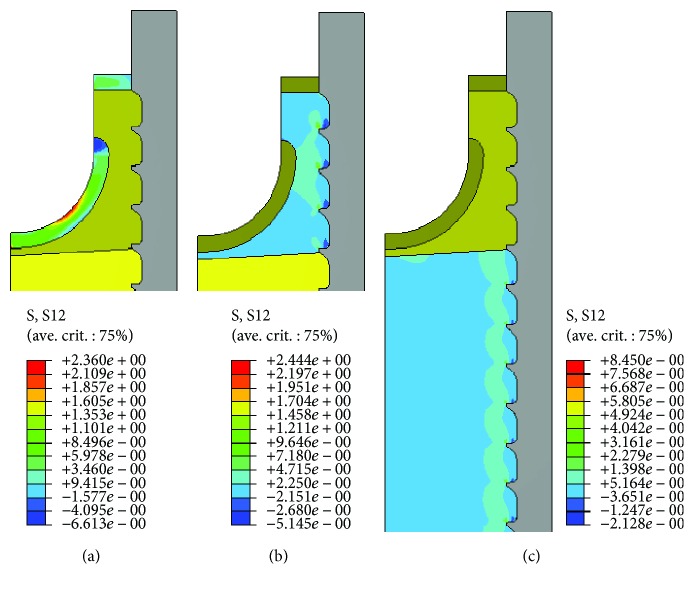
Shear stress distribution computed under small deformation formulation for the N3 insertion in (a) normal cortical, (b) subcortical, and (c) trabecular bone structure.

Figure 6 shows the shear stress distribution for the N3 insertion type using small deformation formulation. Owing to the stiff variability of the bone structures, the shear stress distribution has been displayed for each bone type separately. The various bony components do not behave similarly; the hard cortical bone was found to be more prone to failure under shear stresses while the softer trabecular bone tended to fail less because of its high-energy storage capacity. In the subcortical bone type, the highest shear stresses (~2.4 MPa) have arisen in the tapped areas close to the first thread and have further propagated in the trabecular bone parallel to the loading axis.

### 3.2. Computed Pullout Effort in Normal and Osteoporotic Bone

The ultimate pullout effort is defined as the load for which the pedicle screw leaves its anchorage in the bone. Unlike the loosening mechanism, tearing is often accompanied with unpredictable failure at the bone-implant interface. One of the original inputs implemented in the current study was the incorporation of the subcortical bone while computing the resistive force as a result of the prescribed screw excursion. To our best knowledge, none of the preceding research works had tackled this issue considering only the cortical and trabecular bones.

**Figure 7:**
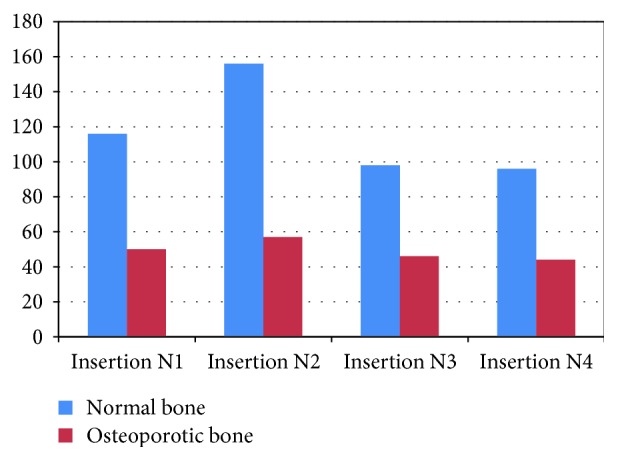
Maximal resistive effort in normal and osteoporotic bone models as a result of a 10 *μ*m screw excursion for various screw insertion types.

In the upcoming analyses, the pullout effort was predicted for the different screw insertion techniques, bone quality types (normal or osteoporotic), and type of loading imposed on the pedicle screw (small and large deformation formulations). Under small deformation formulation, a maximal resistive effort of about 155 N was computed for the N2 insertion case, as shown in Figure 7. The reason for that is that the first screw thread is more deeply engaged in the vertebral subcortical bone, leading to a larger amount of bone being located between the first thread and the cortical bone layer, which substantially enhanced the bone's resistance to the screw pulling out.

**Figure 8:**
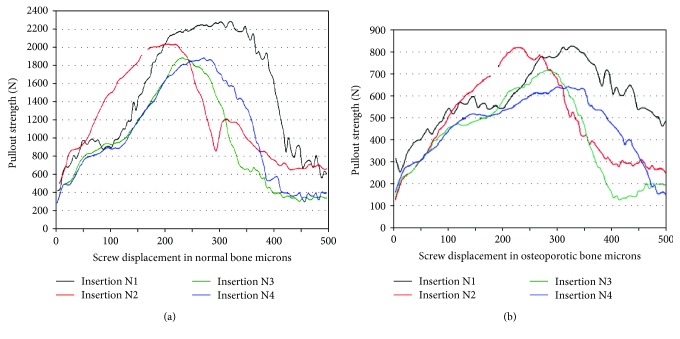
The load-displacement curves under large deformation formulation, plotted for the four types of screw insertion in (a) normal bone and (b) osteoporotic bone.

The load-displacement curves computed under large deformations for the four types of screw insertion techniques, for normal and osteoporotic bones, showed that the N1 insertion technique provided the highest pullout effort (~2300 N) in contrast to the N3 and N4 techniques, supporting each at about 1870 N (see Figures 8(a) and 8(b)). For the osteoporotic bone, similar ultimate pullout strength was recorded for the N1 and N2 insertion techniques (~830 N) while the N4 technique provided the least resistance (~650 N).

As a general observation, the computed results of the pullout effort was highly sensitive to bone density irrespective of the insertion technique with the most drastic loss in the pullout strength of about 65% occurring for the N4 insertion type, characterized by a missing cortical wall. Again, irrespective of the bone density, the N1 technique comes out to be the best screw insertion technique since it provided the highest ultimate strength at the farthest screw displacement along the screw path, as clearly shown in Figures 8(a) and 8(b). These values were close to those established experimentally [7].

In addition to providing an estimation of the screw pullout strength, characterized by the maximal recorded load, the curves predict the overall trend of the resistive forces versus the imposed screw displacement. All curves show an increasing resistive force versus the applied screw excursion until the screw starts to leave its anchorage, known as the ultimate resistive value or pullout strength. The resistive force then decays progressively allowing the screw to slide easier to reach the final prescribed displacement of 500 *μ*m.

The load-displacement curves for an osteoporotic bone generally presented dips that are more pronounced than what have been depicted in normal bone. This may be due to the relatively effortless radial deformation of the bone material freeing, sequentially, the implant threads. Similar irregularities were reported in an in vitro study [22] aimed at estimating the pullout strength of conical and cylindrical screws of 6.5 to 7 mm diameters implanted in cadaveric vertebral pedicles. The authors claim that such irregularities were due not only to the shape of the threads at the bone-implant interface but also to the degree of osseointegration and bonding that has taken place at the interface.

### 3.3. Pullout Efforts as a Function of the Number of Geared Threads

**Figure 9:**
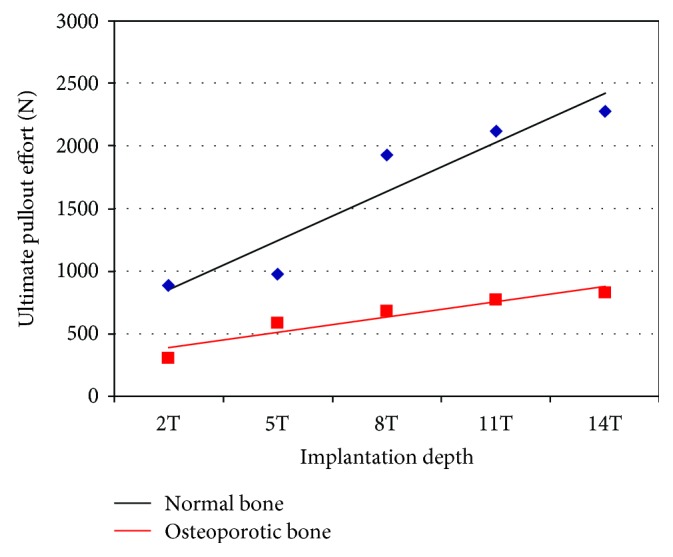
The ultimate pullout effort computed in large deformations as a function of the implantation depth represented by the number of geared threads: two, five, eight, eleven, and fourteen threads (2T, 5T, 8T, 11T, and 14T, resp.).

Figure 9 showed a linear correlation found between the screw ultimate pullout effort and the number of threads engaged under large deformation formulation, while considering the N1 insertion type for both normal and osteoporotic bone types. As the number of threads geared to the bone increases, the axial load induced at the bone-implant interface increased in an almost linear manner for both normal and osteoporotic bone structures; however, this trend was more prominent in normal bone.

The computed ultimate pullout effort on normal bone varied from 900 N for the two-thread-geared model “2T” to 2300 N for the fully inserted-screw model “14T.” During surgeries, the “14T” model is usually adopted to ensure the implant's stability and achieve the desired clinical results. For some pathologies like bone tumor and vertebra congenital deformities, the stability of orthopedic implants in general and the fixation screws in particular become vital. In practice, some extra devices like hooks, transverse connector, and metallic wires are added to the basic instrumentation to guide and relieve the in vivo mechanical loads imposed on the implant to promote its stability. The use of acrylic cement for implants or metallic inserts for mobile implants are used to address the problems caused by osteoporotic bones [23].

### 3.4. Model Validation

Using our finite element model, we considered N1, N2, N3, and N4 insertion techniques and computed the pullout forces for pedicle screws fully inserted in normal bone. The force magnitudes varied from 2300 N for insertion N1 to 1870 N for insertion types N3 and N4. In osteoporotic bone, in contrast, the pullout forces drastically dropped to 830 N for the N1 and N2 and as low as 650 N for the N4 insertion type. The computed ultimate pullout effort for healthy normal bone varied from 900 N for the two-thread-geared model “2T” to 2300 N for the fully inserted-screw model “14T” (Figure 9). We show that in addition to the types and designs of screws used, the surgical technique (insertions) adopted may affect the stability of pedicle screws.

Our computed results are in general agreement with data gathered from experimental studies aiming to measure the pullout strengths under various loading conditions and utilizing selected pedicle screw designs [24–26]. Three groups of screws were used by Yaman et al. [24], namely, the conical cored standard (type A), the dual-threaded (type B), and the dual-cored/dual-threaded (type C) pedicle screws. The recorded mean pullout strength values for these implants tested on bovine vertebrae are 431 N, 614 N, and 752 N, respectively. These screws were further tested on 25 mm and 50 mm thick polyurethane (PU) blocks leading to higher strength values reaching 1050 N for the thickest PU blocks penetrated by the type C device.

Tolunay et al. focused on the usage of dual-lead dual-core with cement augmentation as an alternative to cannulated and standard pedicle screws with cement augmentation [25]. Five groups of pedicle screws, the normal pedicle screw (NPS), the normal pedicle screw with cement (NPS with PMMA), the cannulated pedicle screw with cement (CPS with PMMA), the dual-lead dual-core pedicle screw (DLDC PS), and the dual lead dual core pedicle screw (DLDC PS with PMMA), were designed for this study. Healthy bovine vertebrae and synthetic polyurethane foams, compliant with ASTM F543 standard testing protocols [27], were used as embedding test mediums. The mean pullout values recorded for synthetic foam are 1437 N, 2024 N, 1335 N, 1979 N, and 2895 N while bovine samples provided values of 846 N, 2328 N, 2461 N, 2311 N, and 3917 N for the NPS, NPS with PMMA, CPS with PMMA, DLDC PS, and DLDC PS with PMMA, respectively. These tests showed that PMMA augmentation both before and after screw insertion significantly increases the pullout strength of a pedicle screw (PS). DLDC PS without cement augmentation can provide enough pullout strength as CPS and NPS with PMMA augmentation on healthy animal vertebrae and PU blocks. Considering its high pullout values, DLDC PS with PMMA augmentation could be an alternative to EPS for osteoporotic and severely osteoporotic incidents.

Classical versus newly designed polyether ether ketone expandable shell pedicle screws were biomechanically tested and compared under torsion, pullout, fatigue, flexion, extension, axial gripping capacity, and torsional gripping capacity tests conducted in accordance with ASTM F543 [27], ASTM F1798 [28], and ASTM F1717 [29]. The proposed polyether ether ketone expandable shell pedicle screws are sought to enhance pullout strength, particularly for osteoporotic bone [26]. Indeed, while tests on classical pedicle screws recorded pullout loads of 565 N and 1264 N, the newly proposed system provided 1196 N and 1890 N for PU foam and calf vertebrae, respectively.

## 4. Conclusion

The biomechanical modeling of orthopedic implants is nowadays in high demand since it provides accurate design and assessment of such vital devices. The current study focuses on numerical evaluation of the strength of spinal implants inserted in the pedicle vertebra, since studies that combine both in vivo and numerical simulation through patient-specific models remain very limited.

The current numerical simulation intended to reproduce, as close as possible, some of the physiological solicitations encountered in a normal spine during day-to-day activities. Through this study, the subcortical bone component is implemented in the analysis to better explore the multiscale aspect of the bone. The chosen elastoplastic stress-strain relationships are characteristics of the vertebral bone material and will surely help in the prediction of the pedicle screw-tearing mechanisms.

The accuracy and toughness of the surgical procedure during implant placement can lead to more stable implants. Clinical parameters such as the screw insertion type and depth, the bone density, and the patient degree of mobility greatly affect the mechanisms of the implant's failure/success. As portrayed in this study, the preservation of the pedicle cortex as in the N1 insertion technique and the screw-anchoring depth gearing a maximum number of threads promote better protection against premature breakouts of pedicle screws. The study confirmed the significance of bone density in spine fixation procedures. The pullout strength computed for normal bone drastically dropped in osteoporotic bone. That is, low-density bone characterizing the elderly is found to have most of its pullout strength (~65%) lost with age. The use of acrylic cement and pedicle screws with special hydroxyl apatite coating may strengthen in that case the anchoring sites and improve the pullout strength of such implants.

## Figures and Tables

**Table 1:** Description and value for the parameters used in the 2D CAD model generation.

Parameter	Description	Value (mm)
*L*_1_	Screw length	45
*L*_2_	Screw head length	5
*L*_3_	Pedicle length	5
*L*_4_	Length of the vertebral body specimen	34
*D*_1_	Screw diameter	6
*D*_2_	Pedicle diameter	11
*D*_3_	Diameter of the vertebral body specimen	22
*R*_1_	Pedicle to vertebral body connector	5

**Table 2:** Geometric parameters for the screw design in millimeters.

d1	*Q*	a1	a2	r1	r2	*e*	*P*
6	0.7	3°	35°	0.8	0.3	0.2	2.5

**Table 3:** Mechanical properties adopted for normal and osteoporotic bone structures as well as the titanium alloy used for pedicle screws.

		*ρ* (g/cm^3^)	*E* (MPa)	*ν*	*σ*_p_ (MPa)	*ε*_p_ (%)
Normal bone	Cortical	1.6	12,000	0.3	100	3
	Subcortical	0.5	360	0.3	14.5	20
	Trabecular	0.2	100	0.2	3	40

Osteoporotic bone	Cortical	1	2900	0.3	40	3
	Subcortical	0.3	78	0.3	6	20
	Trabecular	0.13	75	0.2	1.5	40

Pedicle screws	Titanium	—	124,000	0.3	—	—
